# Implication of lipid turnover for the control of energy balance

**DOI:** 10.1098/rstb.2022.0202

**Published:** 2023-10-23

**Authors:** S. Bernard, K. L. Spalding

**Affiliations:** ^1^ Institut Camille Jordan, CNRS, University of Lyon and Inria, Villeurbanne, 69603, France; ^2^ Department of Cell and Molecular Biology, Karolinska Institutet, Stockholm, 17177, Sweden

**Keywords:** obesity, energy-balance model, carbohydrate–insulin model, lipid turnover

## Abstract

The ongoing obesity epidemic is a consequence of a progressive energy imbalance. The energy-balance model (EBM) posits that obesity results from an excess in food intake and circulating fuels. A reversal in causality has been proposed recently in the form of the carbohydrate–insulin model (CIM), according to which fat storage drives energy imbalance. Under the CIM, dietary carbohydrates shift energy use in favour of storage in adipose tissue. The dynamics of lipid storage and mobilization could, therefore, be sensitive to changes in carbohydrate intake and represent a measurable component of the CIM. To characterize potential changes in lipid dynamics induced by carbohydrates, mathematical models were used. Here, we propose a coherent mathematical implementation of the CIM-energy deposition model (CIM-EDM), which includes lipid turnover dynamics. Using lipid turnover data previously obtained by radiocarbon dating, we build two cohorts of virtual patients and simulate lipid dynamics during ageing and weight loss. We identify clinically testable lipid dynamic parameters that discriminate between the CIM-EDM and an energy in, energy out implementation of the EBM (EBM-IOM). Using a clinically relevant two-month virtual trial, we additionally identify scenarios and propose mechanisms whereby individuals may respond differently to low-carbohydrate diets.

This article is part of a discussion meeting issue ‘Causes of obesity: theories, conjectures and evidence (Part II)’.

## 1. Introduction

Obesity is a multifactorial disease, characterized by an energy imbalance, resulting in excess fat storage in adipose tissue. The role of dietary macro-nutrients in the rise of prevalence of obesity has been a topic of intense debate. The calorie-in, calorie-out hypothesis states, in its simplest form, that energy imbalance is independent of dietary composition. There are two fundamentally different ways macro-nutrient composition can impact energy balance. Energy imbalance could be caused by a dysregulation in energy intake (EI). For example, highly processed food with sugar additives could inhibit our sense of satiety, leading to increased EI. In such a case, macro-nutrient composition would have a direct effect on EI, but would not impact on energy expenditure (EE). Another mechanism involves the dysregulation of EE. It has been proposed that high-fat, low-carbohydrate diets lead to higher EE, in addition to lower appetite [1,2]. This evidence depends on methodological aspects, which have been debated [3,4]. When distinguishing between short and long-term trials, a meta-analysis of 29 studies found that EE decreased in the short term but increased in studies with durations longer than 17 days [5]. A recent re-analysis of a large trial indicates that carbohydrate intake, rather than total fat intake, strongly associates with weight loss [6].

The energy-balance model (EBM) proposes that the increased prevalence of obesity results from an excess in food intake and circulating fuels, owing to changes in the food environment [7]. In the EBM, the brain integrates external and physiological signals to control food intake, with energy storage (or mobilization) a consequence of energy imbalance. Dynamic computational models of macro-nutrient metabolism have been developed to explicitly account for interactions between metabolic fuels (dietary changes) and their effect on body composition [8–10]. The carbohydrate–insulin model (CIM) [11–13]) is a recent proposal to synthesize the role of carbohydrates on metabolism through the ratio of insulin-to-glucagon. The CIM differs from the EBM in that the glycaemic load (GL) of a diet is the main driver of energy imbalance [7,11]. In the CIM, a diet with a high GL alters fuel partitioning in favour of fat deposition into adipose tissue. To characterize the CIM against a control model, we consider a simple mathematical implementation of the EBM. The energy in, energy out model (EBM-IOM, [14]) offers a baseline description of the daily EE based on individual physiological characteristics that is compatible with the EBM and satisfies energy conservation.

Under a broad range of physiological conditions, changes in fat mass account for most changes in body weight (BW) [15]. Adipose tissue stores most of the body’s fat in humans, with smaller stores found in the liver and muscle. Energy imbalance, therefore, primarily affects the storage and release of lipids from adipose tissue. Lipid turnover has been measured in humans using radiocarbon dating [16–18] and heavy water strategies [19]. These studies have shown that lipid exchanges are not limited to newly assimilated lipids from food or to energy imbalance, but involve whole adipose tissue stores.

Here, we propose a mathematical and computational implementation of the CIM. This is motivated by two objectives. The first is to test the general coherence of the CIM with respect to its predictions. The second is to see whether the EBM-IOM and the CIM could predict different lipid turnover dynamics, and identify clinical settings that could reveal these differences. Metabolic fuels from food are partitioned among different tissues and between the metabolic pathways leading to storage or oxidation [20]. Energy balance, when viewed statically (without looking for causality or temporality), is determined by a primary energy partitioning between expenditure (oxidation) and storage (which can be negative in case of negative energy balance). There is a second partitioning of the energy allocated for storage among anatomical locations (here, lean and fat tissues). A third partitioning occurs for EE among tissues. In the EBM-IOM, primary partitioning between expenditure and storage is implicitly determined by energy conservation. Anatomical partitioning of energy storage depends on fat mass, according to Forbes equations [15], and anatomical partitioning of EE depends on tissue-specific metabolic rates. A key aspect proposed in the CIM is that macro-nutrient composition alters substrate partitioning between fat deposition and EE, possibly through a combination of mechanisms acting on one or more partitioning factors. Here, we consider the effects of altering the primary or secondary energy partitioning factors. We assume that the third partitioning of EE among tissues is set by biochemical constraints independently of macro-nutrient composition. Carbohydrates could alter the primary substrate partitioning between energy storage and oxidation to favour energy imbalance independently of EI. That is, under most circumstances, a loss of metabolic fuels owing to substrate partitioning will drive increased EI as a primary adaptation, and secondary to this, a change in EE (especially under isocaloric conditions). This is the energy deposition mechanism (CIM-EDM). Additionally, dietary carbohydrates could alter anatomical partitioning of fuel storage to favour accumulation of fat mass instead of lean mass. This is the anatomical partitioning of energy storage mechanism (CIM-APES). In the CIM-EDM, there is no effect of carbohydrate intake on the anatomical partitioning of energy storage. To explore the implications of the different mechanisms, we consider four variants of the CIM: (i) the CIM-EDM, which implements the energy deposition mechanism, (ii) the CIM-APES, which implements the anatomical partitioning of energy storage mechanism, (iii) the combined fuel partition/energy deposition mechanism (CIM-PDM), which implements a combination of the EDM and the APES, and (iv) the full causality reversal mechanism (CIM-REV), which implements a version of the CIM-EDM that does not depend on EI.

**Figure 1.**
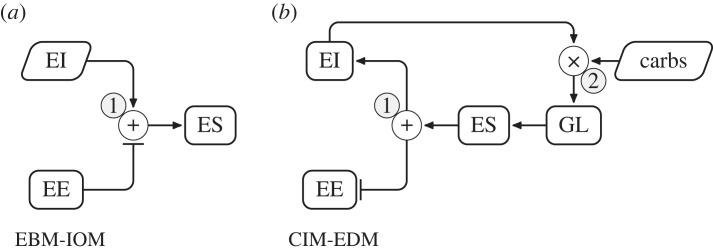
Outline of the causality in the EBM-IOM (*a*) and the CIM-EDM (*b*). EI, energy intake; EE, energy expenditure; ES, energy storage; carbs, per cent carbohydrate intake; GL, glycaemic load. Sharp arrow heads (→) denote a positive action and tee arrow heads (⊣) denote a negative action. Slanted boxes represent an input to the model. Circles represent an interaction between components: additive (+), or multiplicative (×). (1) The ES is equal to the difference between the EI and EE. (2) The EI and the per cent carbohydrate intake determine the GL. See the electronic supplementary material, figure S2 for the complete diagrams of the EBM-IOM and the CIM variants.

We use lipid turnover data obtained from radiocarbon dating over short and long-term time periods [18] to generate two cohorts of virtual subjects and simulate their dynamics according to the EBM-IOM and the CIM variants. Both interpretations of how lipid storage is favoured by dietary carbohydrates, anatomical partitioning of energy storage and energy deposition, were tested, with the energy deposition mechanism most consistent with the CIM. The observed increase in lipid age (corresponding to a slowdown in lipid turnover) in ageing individuals from our clinical data could not be explained by a slowdown in basal metabolism alone, suggesting that additional mechanisms regulate long-term lipid dynamics. During massive weight loss, lipid age did not change significantly under either the EBM-IOM or the CIM-EDM, in line with clinical data ([18]; figure 1*b*), except in a low-carbohydrate diet. Most clinical trials have durations shorter than the 5–15 years used in the virtual cohorts. To assess which aspects of lipid dynamics could provide information on energy balance mechanisms within a more realistic prospective study timeline, we performed a two-month virtual trial. Although the mean lipid age did not change meaningfully over the two-month period, lipid storage dynamics correlated nonlinearly with the extent of weight change in the CIM-EDM. Weight loss was mediated by two different factors. In low-carbohydrate diets, weight loss was facilitated by a high lipid mobilization rate, whereas in high-carbohydrate diets, weight loss was facilitated by a low lipid storage rate. Therefore, virtual subjects with older lipids prior to weight loss tended to lose less weight among those on a low-carbohydrate diet, whereas they lost more (or gained less) weight compared to other subjects on a high-carbohydrate diet. This could explain, under the CIM-EDM, why some individuals respond better to ketogenic (low carbohydrate) diets, better than others.

## 2. Models and methods

The EBM posits that obesity results from an excess in food intake and circulating fuels. Here, we used an energy in, energy out implementation of the EBM (EBM-IOM) presented by Hall *et al.* [14], which describes changes in fat and lean mass in an individual based on a given EI (in kJ d^−1^), physical activity level, age, sex, total fat and lean mass, and carbohydrate intake. The EE (in kJ d^−1^) corresponds to physiological needs; it is computed as the sum of the resting metabolic rate, expenditure related to physical activity, the thermic effect of food, adaptive thermogenesis and the biochemical efficiency of lean and fat masses. Energy storage results directly from the balance between EI and EE (figure 1*a*). The EBM-IOM recapitulates the basic features of the EBM, including energy partitioning, but assumes no active BW regulation via external signals from the environment or from peripheral organs [7]. Details are found in the electronic supplementary material, Models and Methods, §1.1.

To build the CIM, we modified the EBM-IOM with a specific set of hypotheses described by the CIM [11]. We considered several mechanisms for the CIM: CIM-APES, CIM-EDM, CIM-PDM, and CIM-REV. Here, we describe the CIM-EDM, because it generates predictions close to the CIM [11,13]. All mechanisms are detailed in the electronic supplementary material, Models and Methods, §1.2.

As a baseline, we defined EE_50_, or fixed-point EE, as the EE for a medium 50% carbohydrate intake and an average GL. In the CIM-EDM, the effective EE depends on carbohydrate intake, and will differ from the fixed-point EE. The initial value of EE_50_ is set by imposing weight stability, with the associated EI, EI_50_ equal to EE_50_. We have assumed that changes in EE_50_ following changes in EI are described by the EBM-IOM The major hypothesis of the CIM-EDM is that energy storage depends on the GL (figure 1*b*). The GL is the amount of glucose, in grams, that would induce the same glycaemic response as the carbohydrate intake. To capture the effect of the GL, we introduced an energy deposit (ED), defined as the difference between the fixed-point EE and the EE (electronic supplementary material, equation (S21)). We made the hypothesis that the EE decreases with increasing GLs, as more lipid is stored in adipose tissue and not available for expenditure. An EE less than EE_50_ creates a situation where energy is stored while there is a deficit in energy available for expenditure. We took into account the possibility that the ED may elicit an increase in EI, through hunger signals. A parameter *g*_ED_ specifies the extra EI as a fraction of the ED. Because the EE is set, extra EI will be added to storage, leading to weight gain. Conversely, a high EE will induce a mobilization from energy stores, which can feedback to decrease EI, leading to weight loss. Changes in EE were limited to ±10%, based on reported estimates of the effect of switching to a low-carbohydrate diet [21] and the uncertainty of estimating EE outside the laboratory [22].

We expect the EBM-IOM and the CIM variants to modulate lipid storage and mobilization differently. The lipid storage rate is the amount of lipids stored (in kg) per day, and the lipid mobilization rate is the average rate at which lipids are mobilized daily. It is roughly equal to the fraction of the total fat mass mobilized every day for energy needs. The lipid residence time (or lipid age) is the mean time lipids spend in the adipose tissue before mobilization for energy needs. At energy balance, lipids are exchanged 1-to-1, and this is termed the lipid turnover rate. Lipid age is roughly equal to the inverse of the lipid turnover rate, although when not at steady-state (during changes in fat mass) may also depend on lipid storage rates. The lipid age is an interesting marker for validating the CIM, as it can be measured in clinical settings [18]. However, energy conservation only provides loose constraints on lipid storage and mobilization rates. In order to track lipid age, we made the additional assumption, common to all models, that the lipid storage rate correlates with the total EI. Once the lipid storage rate is set, the lipid mobilization rate can be computed by balancing the energy storage equations. The full model is completely specified by a set of dynamic variables governed by ordinary differential equations, a set of individual parameters and a set of initial values for the dynamic variables. A virtual subject is an instance of the model with specific individual parameters and initial values. Biochemical constants and other parameters common to all virtual individuals are listed in the electronic supplementary material, table S1). The model has six dynamic variables (fat mass, lean mass, glycogen stores, adaptive thermogenesis, GL and lipid age). Other quantities of interest, such as EE or the lipid storage and mobilization rates, can be computed from the dynamic variables.

We constructed a longitudinal dataset by numerically solving the model for a cohort of virtual subjects (a simulation). The initial values specify the state of each subject at the beginning of the simulation. In all simulations, we set the initial values of the dynamic variable to their equilibrium values. In particular, this means that virtual subjects were assumed to be weight stable before the simulation (as was the case of the clinical cohort data used to generate the virtual patient cohorts).

**Figure 2.**
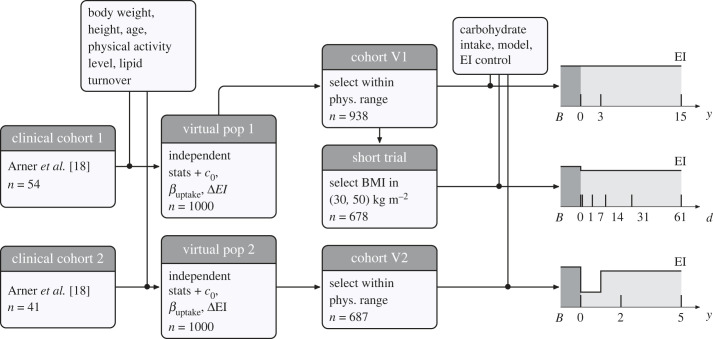
Generation of virtual cohorts. Two virtual populations were generated based on clinical cohort statistics. Each virtual subject was generated randomly, with uncorrelated physiological parameters. Two virtual cohorts were set up by selecting subsets of virtual subjects with physiological parameters within clinical ranges. The short trial cohort is a subset of cohort V1 with BMI values between 30 and 50 kg m^−2^. At baseline, each virtual individual is assumed to be weight stable under a medium carbohydrate diet, with EI = EE = EE_50_.

We generated two virtual populations based on two previously published clinical cohorts [18], in which lipid turnover rates were followed during ageing (cohort 1, for 15 years) and weight loss (cohort 2, for 5 years). For each cohort, we generated 1000 virtual female subjects with independent normally distributed values of initial BW, height, age, physical activity level, baseline dietary carbohydrate intake fraction, and beta distributed parameters controlling the rate of lipid storage (figure 2; electronic supplementary material, tables S2 and S3). Means and standard deviations of BW, height, age and physical activity level were taken to match clinical data. Pre-existing physiological correlations in the clinical cohorts were removed by the generation process. This means that the physiological parameters in each virtual individual are uncorrelated, reducing the risk for confounding factors in the simulations. Some of the subjects generated by this process had non-physiological characteristics, such as combinations of BWs and heights leading to extreme body mass index (BMI) values. Virtual subjects with BW, height or physiological activity level falling outside the range of clinical data were removed. The remaining virtual subjects were assigned to cohort V1 (*n* = 938), and cohort V2 (*n* = 687). The two virtual cohorts were used to explore the consequences of carbohydrate intake on long-term (5–15 years) lipid dynamics. A virtual trial was set up to look at short-term changes (over two months) in lipid dynamics following moderate weight loss. The virtual trial used a subset of obese subjects from cohort V1 (*n* = 678). Cohort statistics are in the electronic supplementary material, table S4.

The virtual longitudinal datasets were generated by numerically simulating the virtual cohorts with different parameters, with the EBM-IOM or the CIM variants. For the CIM-EDM, simulations were performed with (*g*_ED_ = 1) or without (*g*_ED_ = 0) a feedback loop on EI. For each simulation, all virtual subjects were set to a baseline value where they are weight stable. During each simulation, one or more parameters were changed to mimic the effects of an intervention. In cohort V1, the EI and carbohydrate intake fraction were modified. The EI was modified from baseline in each subject by a random amount normally distributed with a zero mean and a standard deviation of 100 kJ d^−1^. Simulations were then repeated with carbohydrate intake set to fixed values ranging from 10% to 90%, in steps of 10%. In cohort V2, the EI in each virtual subject was adapted to reproduce massive weight loss during the first year, followed by a weight stable or weight rebound period, similar to clinical data [18]. Simulations were repeated with carbohydrate intake set to fixed values ranging from 10% to 90%, in steps of 10%. Changes in parameter values have a transient effect on lipid storage and mobilization rates. For that reason, for these variables, we report baseline physiological values before changes in parameters and not values at year 0, after parameters have been changed.

## 3. Results

### (a) Cohort V1—ageing (15 years)

**Figure 3.**
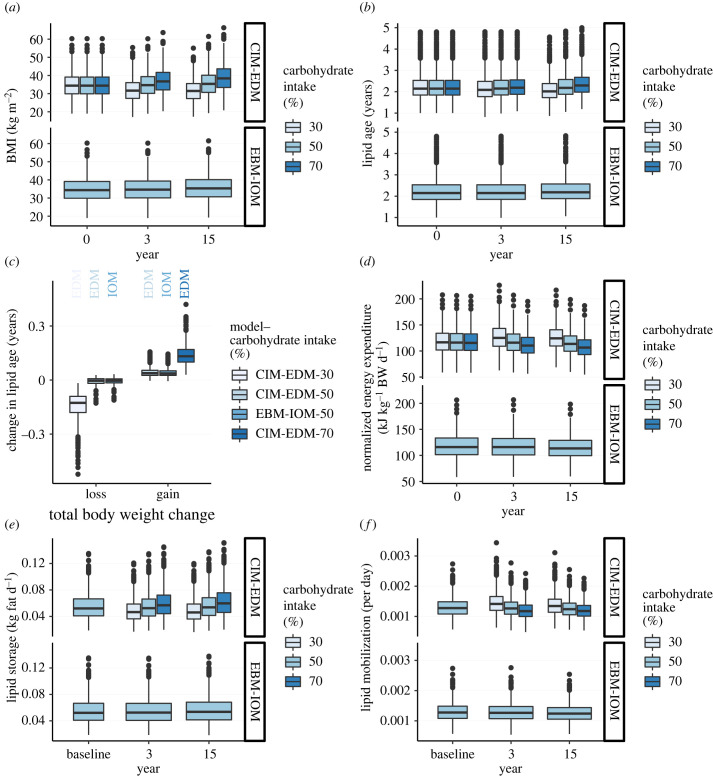
Cohort V1—ageing, CIM-EDM and EBM-IOM. (*a*) BMI distribution at year 0, year 3 and year 15. (*b*) Lipid age at year 0, year 3 and year 15. (*c*) Change in lipid age in weight loss or weight gain (difference in body weight (BW) between year 15 and year 0). (*d*) Normalized EE (EE/BW, in kJ kg^−1^ BW d^−1^) with respect to carbohydrate intake at year 0, year 3 and year 15. (*e*) Lipid storage at baseline, year 3 and year 15. (*f*) Lipid mobilization rate at baseline, year 3 and year 15. See the electronic supplementary material, figure S6 for CIM variants.

In the CIM-EDM, carbohydrate intake has an impact on energy balance, with lower carbohydrate intake leading to lower values of BMI, and higher carbohydrate intake to higher BMI values (figure 3*a*, CIM-EDM). In the EBM-IOM, the BMI distribution did not change notably between year 0 and year 15, although it slightly increased owing to a lower resting metabolic rate with ageing. A reduced resting metabolic rate with ageing could affect lipid turnover. With a medium carbohydrate intake, there was no change in lipid age over time. With a low carbohydrate intake, lipid turnover increased (lipid age decreased), and with a high-carbohydrate intake, lipid turnover decreased (figure 3*b*, CIM-EDM). When broken into tertiles of total BW change, changes in lipid age were mostly associated with low and high-carbohydrate intakes. With a medium carbohydrate intake, a correlation was present in the EBM-IOM and the CIM-EDM, but to a lower extent (figure 3*c*). The CIM-EDM predicts that carbohydrate intake affects EE independently from changes in BW. To control for changes in BW, we compared the EE normalized for total BW (EE/BW; figure 3*d*). With a low-carbohydrate intake (30%), the normalized EE increased between year 0 and year 15, while it decreased with a high-carbohydrate intake (70%). With medium carbohydrate intake (50%), the change was negligible. The lipid storage rate correlated to the carbohydrate intake in the CIM-EDM at years 3 and 15 (figure 3*e*). However, a low-carbohydrate intake was associated with an important increase in lipid mobilization rate over years, while a high-carbohydrate intake was associated with a decrease in lipid mobilization rate (figure 3*f*).

### (b) Cohort V2—weight loss (5 years)

**Figure 4.**
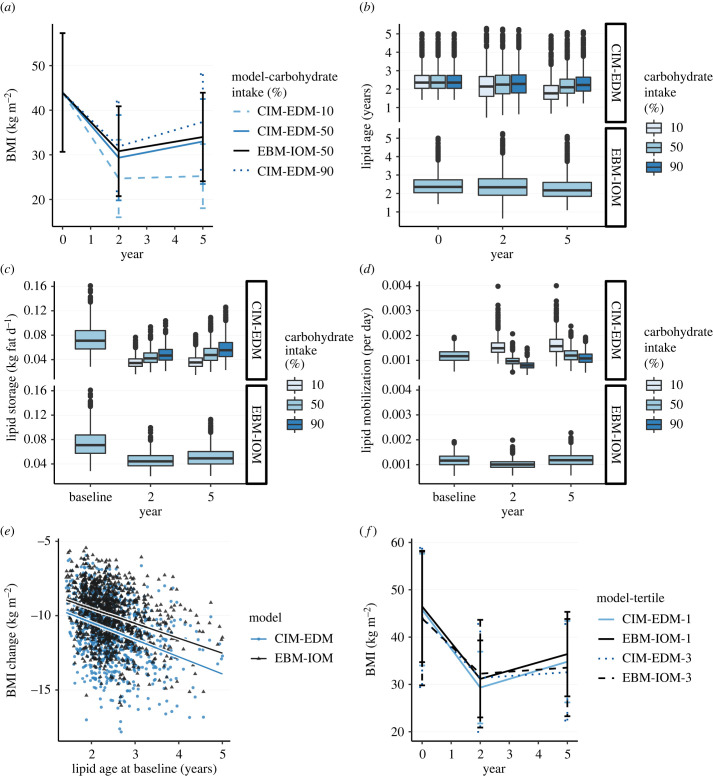
Cohort V2—weight loss, CIM-EDM and EBM-IOM. (*a*) BMI at year 0, year 2 and year 5. (*b*) Lipid age at year 0, year 2 and year 5. (*c*) Lipid storage at baseline, year 2 and year 5. (*d*) Lipid mobilization rate at baseline, year 2 and year 5. (*e*) Change in BMI between year 0 and 5, versus lipid age at baseline, with regression lines. (*f*) BMI at year 0, year 2 and year 5, for the first (1) and third tertile (3) of body weight change between year 2 and year 5. Tertile 1 means most weight gain. In (*e*) and (*f*), carbohydrate intake is 50%. See the electronic supplementary material, figure S2 for the CIM variants.

Following a restriction on EI to 3766 kJ d^−1^ for the first nine months, the mean BMI decreased in all carbohydrate conditions at year 2. However, the mean BMI at year 5 ranged from almost lean (26 kg m^−2^) in the low-carbohydrate intake group, up to a pre-weight loss level in the high-carbohydrate intake group (39 kg m^−2^ at year 5 versus 44 kg m^−2^ at baseline; figure 4*a*). Lipid age remained approximately constant in all conditions except in the low-carbohydrate intake group, where the mean lipid age significantly decreased (figure 4*b*). There was no difference in lipid age between the medium and high-carbohydrate intake groups or the EBM-IOM. The lipid storage rate was strongly associated with carbohydrate intake. Compared to baseline, the lipid storage rate at year 5 decreased most with low-carbohydrate intake, while there was a moderate decrease with high-carbohydrate intake (figure 4*c*). With a low-carbohydrate intake, changes in lipid mobilization rates from baseline were pronounced at years 2 and 5 (figure 4*d*). With medium or high-carbohydrate intake, lipid mobilization rates decreased near or below baseline values at years 2 and 5. Consistent with clinical data [18], total change in BMI between years 0 and 5 negatively correlated to baseline lipid age with medium carbohydrate intake, in the CIM-EDM and the EBM-IOM, even though the CIM-EDM was associated with a greater BMI decrease (figure 4*e*). Similar results were obtained in simulations with other carbohydrate intake levels (not shown). When grouped in tertiles of the largest BW changes between year 2 and 5, differences in the CIM-EDM were explained by BMI differences at year 2, while differences in the EBM-IOM were explained by BMI differences at year 5 (figure 4*f*).

### (c) Two-month virtual trial

To explore the potential role of lipid turnover in the CIM-EDM and the EBM-IOM over a clinically relevant time interval, we simulated cohort V1 over two months under an energy restricted diet with 10% (low), 50% (medium) and 90% (high) carbohydrate intake, under controlled, isocaloric EI (*g*_ED_ = 0, no EI feedback loop), or uncontrolled EI (*g*_ED_ = 1, virtual subjects change their EI according to their ED). We selected cohort V1 subjects with a BMI between 30 and 50 kg m^−2^, for a total of *n* = 678 subjects. Parameters are as in the electronic supplementary material, table S2, except that ΔEI = −1000 kJ d^−1^.

**Figure 5.**
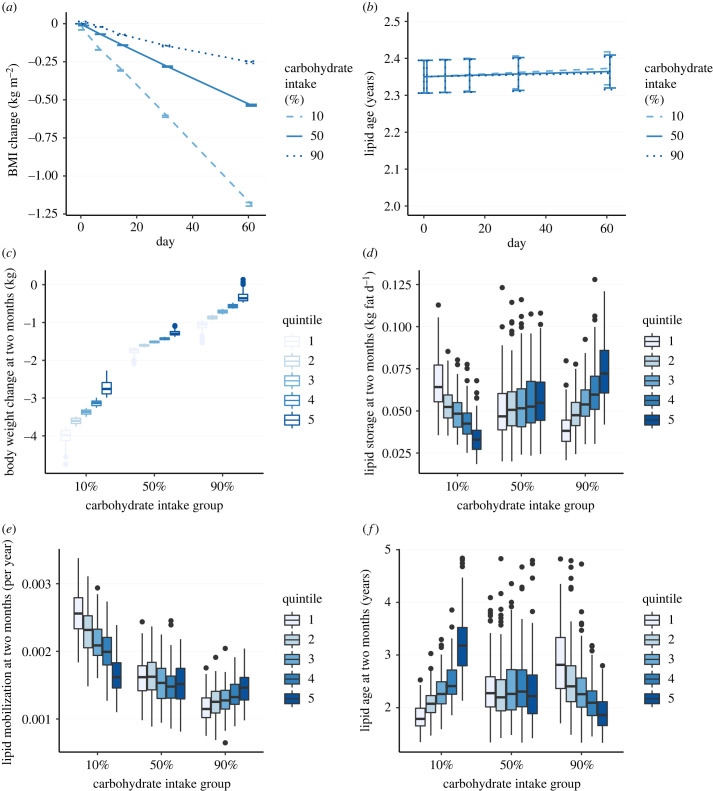
Two-month virtual trial, CIM-EDM, uncontrolled energy intake (*g*_ED_ = 1). In (*c*–*f*), the grouped boxplots correspond to quintile 1 (left, most weight loss) to 5 (right less weight loss). (*a*) BMI over two months (error bars are standard errors). (*b*) Lipid age over two months (error bars are standard errors). (*c*) Total body weight change at two months. (*d*) Lipid storage at two months. (*e*) Lipid mobilization rate at two months. (*f*) Lipid age at two months. See the electronic supplementary material, figure S8 for CIM variants.

Total BW, lipid storage and mobilization rates and lipid age in the uncontrolled intake simulations (*g*_ED_ = 1) showed that the mean BMI decreased significantly more in the low-carbohydrate intake group than in the medium or high-carbohydrate intake groups (figure 5*a*). Lipid age remained unchanged during the two-month interval (figure 5*b*). The cohort was broken into quintiles of total BW loss, with rank 1 corresponding to most weight loss. Marked differences in BW change were seen across quintiles in the low-carbohydrate intake group, compared to the medium or high-carbohydrate intake groups (figure 5*c*). The lipid storage rate did not vary significantly in the medium carbohydrate intake group across quintiles; however, it was strongly correlated to weight loss in the low and high-carbohydrate intake groups (figure 5*d*). Interestingly, in the low-carbohydrate intake group, weight loss was associated with high lipid storage rates (figure 5*d*; 10% carbohydrate intake). Lipid mobilization followed the same correlation patterns with respect to weight loss. With a low-carbohydrate intake, the decrease in BW correlated significantly with an increase in lipid mobilization (figure 5*e*; 10% carbohydrate intake). A similar, although less pronounced relationship was seen for BW change and lipid mobilization for moderate carbohydrate subjects (intake 50%). Interestingly, in the high carbohydrate intake group, lower lipid mobilization was associated with more weight loss (figure 5*e*). Although lipid age remained constant over time, there was a strong effect on weight loss within each carbohydrate intake group. In the low-carbohydrate intake group, lipid age at two months correlated negatively with weight loss (younger lipids associated with more weight loss). However, in the high-carbohydrate intake group, the effect was opposite, with older lipids associated with greater weight loss (figure 5*f*). In the medium carbohydrate intake group, there was no effect of lipid age. Similar results were obtained with the isocaloric intake simulations (electronic supplementary material, figure S3, *g*_ED_ = 0).

## 4. Discussion and conclusion

We have presented an implementation of the CIM based on the EDM that includes a component for lipid storage dynamics. The CIM-EDM is consistent with the reversal of causality hypothesis that energy storage determines energy balance. Simulation results from virtual cohorts show a broad agreement with the main predictions of the CIM-EDM about the effect of dietary carbohydrates on BW changes. We also explored other mechanisms related to the CIM: CIM-APES and CIM-REV. In the CIM-APES, dietary carbohydrates promote the deposition of extra energy as fat. The partitioning parameter was allowed to vary between 40% and 160% of the value set in the EBM-IOM. The CIM-APES was in itself not consistent with a reversal of causality, because of the need for a CIM-independent way to determine the energy imbalance (electronic supplementary material, figure S9). Moreover, the CIM-APES does not provide an explicit mechanism for fat accumulation, and an additional mechanism is needed for fat accumulation under a high dietary carbohydrate intake. This is inconsistent with the CIM prediction that macro-nutrients have an effect even in isocaloric diets [11]. The CIM-APES may also lead to unrealistic or inconsistent changes in lean mass. In the short trial, the CIM-APES predicts a greater BW loss with a high-carbohydrate diet, owing to loss of lean mass. In cohort V2, after weight loss, some of the individuals saw a gain in lean mass (electronic supplementary material, figure S9*a*, CIM-APES, negative points). The CIM-REV provides a full reversal in causality, in that only the GL, and not the total EI, determines energy storage. While the results are similar to those obtained with the CIM-EDM, the CIM-REV is insensitive to increased food intake owing to hunger, as long as the GL remains unchanged, leading to an attenuated response to changes in carbohydrate intake compared to the CIM-EDM (electronic supplementary material, figure S8). We suggest that the CIM-EDM best reflects the key features of the CIM in providing an explicit mechanism for the reversal of causality between energy imbalance and energy storage, while maintaining a sensitivity to changes in food intake.

### (a) Dynamics of lipid storage and mobilization

Remarkably, there was almost no impact on lipid age dynamics when considering medium or high-carbohydrate intake groups. In cohort V1, a high-carbohydrate intake led to an average increase in lipid age of less than 0.25 years compared to a medium carbohydrate intake (figure 3*b*). This difference is unlikely to be visible in clinical conditions. By comparison, lipid age dynamics were affected by a low-carbohydrate intake. In a low-carbohydrate diet, increased daily energy needs are met by lipolysis from adipose tissue rather than from dietary sources. Consequently, old lipid stores become quickly depleted, as measured by lipid mobilization rates, leading to a decrease in lipid age (figure 3*f*). By contrast, high-carbohydrate intake levels were not associated with older lipids. This might be surprising at a first glance, but a high-carbohydrate intake is associated with higher lipid storage and lower lipid mobilization rates. These two factors push the lipid age in opposite directions: there are more new lipids, but old lipids stay longer. Regardless of changes in lipid age over time, lipid turnover is a factor associated with BW change. In cohort V2, a decrease in BMI was correlated with lipid age at baseline, such that a lower lipid age at baseline correlated with a smaller decrease in BMI (figure 4*e*). This observation is puzzling, and was already noted in the clinical cohort 2 [18]. We can use the computational model to provide a mechanistic explanation for this observation. The lipid age at baseline is given by electronic supplementary material, equation (S30). It is inversely correlated to the lipid storage rate (electronic supplementary material, figure S4), meaning that individuals with older lipids have a low lipid storage rate, and accordingly, are more prone to weight loss. The original interpretation was that a low lipid mobilization rate would open a window of opportunity for adaptation [18, p. 1388].

A long term increase in lipid age was observed in clinical cohort 1, and was associated with a slower mobilization rate rather than a lower lipid storage rate. We could not reproduce a significant age-associated increase in lipid age in cohort V1. There was a significant increase in lipid age with higher carbohydrate intake. This, however, was associated with an increase in BMI, something that was not observed in clinical cohort 1 (fig. 2*c* in [18]). These results suggests the presence of an additional age-related mechanism, besides a change in metabolic rate, contributing to the observed decrease in lipid turnover in our clinical cohort [18]. A decline in lean mass, owing to a decrease in physical activity with ageing, could contribute to a change in lipid turnover rates.

Lipid mobilization is a consequence of energy balance (electronic supplementary material, equation (S1)). The lipid mobilization rate can be estimated in weight stable individuals by radiocarbon dating [23], but has limits during weight change [16]. Here, we calculate lipid mobilization rates directly from numerical simulations, by using the electronic supplementary material, equation (S31). This provides an unbiased picture of the dynamics of lipid mobilization during weight loss. In the CIM-EDM, lipid mobilization rates were affected by carbohydrate intake. In cohort V1, in the low-carbohydrate intake group, mobilization rates were slightly elevated compared to medium carbohydrate intake regimens. In the high carbohydrate intake group, mobilization rates decreased slightly at years 3 and 15. In cohort V2, lipid mobilization rates followed the same general trends. Thus, data obtained from our virtual patient cohort with the CIM-EDM supports the notion that dietary carbohydrate content impacts the rate of lipid mobilization, such that higher carbohydrate content decreases lipid mobilization from white adipose tissue and lower carbohydrate content increases lipid mobilization.

In the two-month trial, the lipid age did not significantly change over time, but was strongly associated with weight loss in the low and the high-carbohydrate intake groups (figure 5*f*). Interestingly, the correlation between weight loss and lipid age was opposite in the low and the high-carbohydrate intake groups. These results provide a non-trivial prediction of the CIM-EDM on lipid dynamics in low and high-carbohydrate intake diets during weight change. In low carbohydrate diets, the CIM-EDM predicts that weight loss is facilitated by a high lipid turnover rate, whereas in high-carbohydrate diets, weight loss is predicted to be facilitated by a low lipid turnover. Therefore, subjects with older lipids are expected to lose less weight among those on a low-carbohydrate diet, whereas they lose more weight compared to other subjects on a high carbohydrate diet. These predictions are based on lipid age before dietary intervention, suggesting that the baseline phenotype of an individual (high or low lipid turnover) could determine the outcome of a diet based on a change in carbohydrate content, such as a ketogenic diet. In a large clinical cohort, lipid turnover rates were lower in overweight and obese individuals [16]. Together with CIM-EDM simulation results, this suggests that low lipid turnover rates in obesity could impede low carbohydrate weight loss strategies. Along the same lines, lean individuals with a high lipid turnover rate could be more prone than overweight or obese individuals to gain weight with a high-carbohydrate diet. These observations could explain why some people respond better than other to specific diets. In addition to metabolic status, age and sex could be important factors to take into account for adapting macro-nutrient composition [24].

These predictions could be tested in a short-term four- or eight-week trial in individuals with overweight or obesity with average GL diets. Lipid turnover rates could be assessed at baseline, followed by a very low-carbohydrate or ketogenic diet for at least three weeks. The CIM-EDM predicts that the lipid turnover rate at baseline would correlate with the extent of weight loss.

### (b) Energy deposit and control of lipid storage

According to the CIM-EDM, a lower GL is associated with increased EE, promoting weight loss. However, weight loss would also result in a reduction of EE related to BW, so it is not clear whether EE would be sustained during weight loss. Indeed, the EE decreased in low, but increased in high-carbohydrate intake regimens (electronic supplementary material, figure S5). When normalizing for BW; however, the EE increased in low, but decreased in high-carbohydrate intake regimens (figure 3*d*). Body mass changes might, therefore, mask an increase in EE [5,6,21]. In the short trial, after two months of weight loss, for each 10% decrease in carbohydrate intake, the normalized EE increased by 1.4 kJ d^−1^ kg BW^−1^. This corresponds, for a BW of 100 kg, to 140 kJ d^−1^ (33 kcal d^−1^), a value similar to what was reported in a meta-analysis [5]. After two months, the normalized EE per kg BW increased by 4.8% with a low-carbohydrate diet, but decreased by 1.5% with a high-carbohydrate diet.

The introduction of the ED reverses causality on EE. In the EBM, all fuel from food is made available for metabolism (figure 1*a*), whereas in the CIM, primary substrate partitioning drives the access of metabolically active tissues to fuel (figure 1*b*). Although this is a major departure from the EBM paradigm, the EBM could also admit a limit on fuel availability. Maximal human EE from alimentary sources has been estimated at about 2.5 times the basal metabolic rate (BMR), but total EE could go up to almost 10 times the BMR [25]. These figures suggest that humans can sustain, over short periods of time, an energy imbalance of up to 7.5 times the BMR. There is also a limit on lipid mobilization to about 0.7% of the fat mass per day [26]. We have estimated lipid mobilization rates to be around 0.002 d^−1^. For a fat mass of 50 kg, as in cohort V2 at the beginning of weight loss, this amounts to $0.1\;{\mathrm{kg}\;d}^{-1}$ of lipids. The theoretical limit for the same cohort is 0.350 kg d^−1^ (0.07% d^−1^ × 50 kg fat mass). Therefore, even during initial weight loss, lipid mobilization is below the theoretical limit, indicating that under most conditions, the models used here operate within physiological bounds.

### (c) Limitations

Recent discussions have addressed the clinical and biological evidence in support and against the CIM [7,11,12,27]. Our modelling approach has several limitations in informing this debate; notably there a risk of oversimplifying the conceptual models. Here, we focused on alterations in fuel partitioning and storage and their impact on lipid dynamics. Hormonal regulation of metabolism, gut-brain axis or other physiological controls are not explicitly taken into account.

There is evidence that EE is greater on a low-carbohydrate diet during weight maintenance [2]. Concerns have been raised that post-, rather than pre-weight loss EE was used as baseline [3]. BW composition and metabolism changes during weight loss, however, argue for using the post-weight loss baseline [4]. Our simulations of cohort V1 predict a smaller absolute EE, but a greater normalized EE per kg BW after weight loss on a low carbohydrate diet (figure 3*d*; electronic supplementary material, figure S5), suggesting that the choice of the baseline is important. In another study, a ketogenic diet was accompanied by a modest increase in EE [28], but a re-analysis found a larger increase in subjects not confined to metabolic chambers [21]. The CIM also predicts a reduced EI on a low-carbohydrate diet. A recent study on the effect of plant-based diets found that EI was greater in the low-carbohydrate diet group than those from the low fat diet group after two weeks [29]. It has been suggested, however, that the metabolic effects of low-carbohydrate diets appear after more than two weeks [5]. Our implementations of the CIM did not include a mechanism for transient adaptation to low or high-carbohydrate diets, but fuel partitioning could play a role, as suggested by the opposite trend of the CIM-APES in BW loss in the short trial (electronic supplementary material, figure S8*a*). We made no assumptions regarding the allocation of extra EE, which could be related to voluntary exercise, resting EE, muscle work efficiency or thermogenesis [13,21]. We make no claim regarding the biological plausibility of an ED, for a discussion of the evidence, see [3,5,12,29]. Part of the results obtained here are based on nonlinear rates in the electronic supplementary material, equations (S18) and (S23). These terms have not been validated by independent data, but we tried to remain within physiological bounds.

Formal model implementations, mathematical or numerical can be useful for validation studies, where hypothesis testing requires accurate predictions. The CIM proposes that lipid storage is modulated by dietary carbohydrates. We identify clinically testable lipid dynamic parameters that discriminate between the CIM-EDM and the EBM (EBM-IOM). Using a clinically relevant two-month virtual trial, we additionally identify scenarios and propose mechanisms whereby individuals may respond differently to low carbohydrate diets.

## Data Availability

The numerical implementation of the model, the scripts to generate the datasets and the scripts to generate the tables and figures have been deposited at https://gitlab.inria.fr/bernard1/ebm-cim-lipid. The data are provided in the electronic supplementary material [30].
